# Health inequalities in Germany and in international comparison: trends and developments over time

**DOI:** 10.17886/RKI-GBE-2018-036

**Published:** 2018-03-20

**Authors:** Thomas Lampert, Lars Eric Kroll, Benjamin Kuntz, Jens Hoebel

**Affiliations:** Robert Koch Institute, Berlin, Department of Epidemiology and Health Monitoring

**Keywords:** HEALTH INEQUALITIES, SOCIOECONOMIC STATUS, TREND ANALYSES, HEALTH MONITORING

## Abstract

Social epidemiological research has consistently demonstrated that people with a low socioeconomic status are particularly at risk of diseases, health complaints and functional limitations, and die at younger ages than those with a higher socioeconomic status. Greater stresses and strains in the workplace, family and living environment are under discussion as possible explanations. Health-related behaviours, psycho-social factors and personal resources, which are important in coping with everyday demands, certainly also play a role. From a public health and health policy perspective, reducing these health inequalities is an important goal. Insights into developments and trends in health inequalities over time can contribute towards highlighting new and emerging problems, and can thus help identify possible target groups and settings for relevant interventions. At the same time, these insights provide a basis upon which the success of policies and programmes that have already been implemented can be analysed and measured. Against this background, this review examines how health inequalities in Germany have developed over the last 20 to 30 years and places its findings within the context of the latest international research in this field.

## 1. Introduction

Inequalities in health and life expectancy linked to socioeconomic status – also referred to as health inequalities – are a central issue in public health and health policy [1-3]. These days, the social and political relevance of health inequalities is widely discussed and debates also focus on the economic and ethical aspects of these issues [4]. Moreover, this debate is increasingly concerned with the question of what can be done to improve the health of the entire population and to reduce health inequalities in the long term [5, 6]. Answering this question has become all the more urgent, since experience gained in recent years has shown that many of the measures and interventions that have already been implemented, especially in prevention and health promotion, have not been able to sufficiently reach socially disadvantaged groups [7, 8]. Furthermore, observations have shown that health inequalities can be reproduced even in the wake of social changes [9]. However, it is still unclear exactly how current developments, such as the expansion and spread of poverty into wider sections of society and the rise of precarious employment, affect the extent and manifestation of health inequalities [10].

**Info box: Absolute and relative health inequalities**

Analyses of social inequalities in health and life expectancy often distinguish between absolute and relative health inequalities. Absolute health inequalities are typically quantified using rate differences between the lowest and highest socioeconomic groups. In contrast, relative health inequalities are calculated using the rate ratios that exist between these groups. Research suggests that a reduction of absolute health inequalities occurs under a wider range of conditions than a reduction of relative health inequalities [16]. If risks or prevalence rates within a population decline overall, relative inequalities may increase, whereas absolute inequalities may remain constant or even decrease during the same period [17, 18]. As such, the trends found and conclusions drawn in a particular study can largely depend on whether absolute or relative health inequalities are considered. Therefore, it is recommended to take both absolute and relative inequalities into account when analysing changes in health inequalities over time [19-22].

The fact that social inequalities in living conditions and social inclusion are reflected in health and life expectancy has been sufficiently documented [2, 11, 12]. Every year, numerous studies demonstrate that people with a low socioeconomic status – mainly measured using data on education, occupation and income [13, 14] – are more often exposed to health burdens and risks in areas such as the workplace, the home and their living environment than people with a higher socioeconomic status. The same applies to behavioural health risk factors, such as smoking, physical inactivity and an unhealthy diet. These inequalities are compounded by the fact that people with a low socioeconomic status have fewer social and personal resources, which are beneficial to health as they help people cope with the demands and pressures they face. As such, many illnesses, health complaints and functional limitations are more prevalent among people with a low socioeconomic status. Moreover, people with a low socioeconomic status also provide worse self-assessments of their own health and their health-related quality of life than people with a high socioeconomic status. The increased prevalence of health risks and diseases among people in the lowest socioeconomic group is ultimately reflected in a lower life expectancy; in Germany, the people in the highest socioeconomic group have a five to ten year longer mean life expectancy at birth than people in the lowest socioeconomic group [15].

Planning and implementing policies and programmes aimed at reducing health inequalities requires data and analyses that provide insights into how social inequalities in health and life expectancy develop over time. On the one hand, these insights are important because they help identify relevant developments at an early stage and can point to new or emerging problems. Moreover, they help to determine possible target groups and relevant settings for interventions. On the other hand, the careful analysis of trends and developments over time provides an essential basis upon which to assess and review policies and programmes that have already been implemented.

Unlike in the UK, the US and Scandinavian countries, for a long time analyses of trends and temporal developments in health inequalities were only possible to a limited extent in Germany. This was due to the fact that the data varied in informative value and availability. In recent years the data has improved significantly, and this has opened up new research perspectives. This was supported by the establishment of the German national health monitoring system at the Robert Koch Institute (RKI), which in turn emerged from the German Cardiovascular Prevention Study (DHP) conducted in the mid-1980s and the German National Health Interview and Examination Survey 1998 (GNHIES98). Other health surveys and epidemiological studies, some of which have a narrower thematic focus, now enable more long-term observations to be made using data from repeated cross-sectional surveys, such as the Epidemiological Survey of Substance Abuse (ESA), the National Food Consumption Study (NVS) and the German Oral Health Study (DMS). The German Socio-Economic Panel (SOEP), the German General Social Survey (ALLBUS) and other social science surveys also provide additional databases. These are complemented by official statistics – in particular from the German microcensus and the European Statistics on Income and Living Conditions (EU-SILC) – as well as by data collected from social insurance agencies that are increasingly being used for epidemiological research and health monitoring.

The aim of this review is to describe time trends in health inequalities in Germany over the past 20 to 30 years using published research findings. Moreover, this article also describes the dynamics that underlie these developments. For example, widening health inequalities may be related to the fact that positive developments are only or increasingly observed among population groups with a high socioeconomic status or a relatively advantaged social position. At the same time, negative developments among socially disadvantaged populations may explain why health inequalities are widening. This article focuses on the middle-aged population (25-to 69-year-olds), as developments in health inequalities in childhood, adolescence and old age require a separate consideration which takes the specificities of each life phase into account. The review begins by giving an outline of the research findings available for Germany, before placing these results within the context of the latest international research. However, due to the large number of studies focussing on time trends in health inequalities, this paper can only describe a selection of relevant examples.

## 2. Research findings from Germany

**Figure 1:**
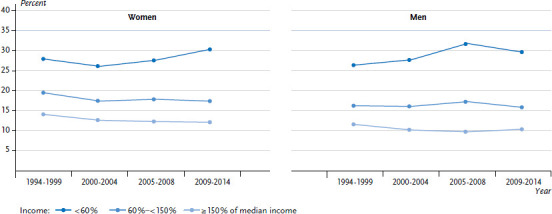
Time trends in self-rated general health (‘fair’ or ‘bad’) among women and men aged 25 to 69 years by income level (age-standardised to the 2013 European standard population) Source: SOEP 1994-2014

Analyses of time trends in health inequalities often refer to self-rated health, an indicator reflecting the subjective dimension of health and well-being. Longitudinal studies have shown self-rated health to be an independent predictor of the uptake of health services and further life expectancy [23, 24]. According to data taken from the SOEP, between 1994 and 2014 a widening of income inequalities was observed for self-rated health. The proportion of women and men with middle and high incomes who rated their overall health as ‘fair’ or ‘bad’ decreased slightly during this period; however, it increased among women and men with low incomes. A comparison of the figures from the first and last observation period demonstrates that absolute income inequalities in self-rated health widened by 2.4 percentage points among women and by 3.2 percentage points among men (Figure 1).

Another recent study that focused on the same observation period, but differentiated between population groups with different levels of education among 30- to 49-year-olds, reached a somewhat different conclusion [25]. The study identified pronounced educational inequalities in self-rated general health over the entire observation period. Over time, the extent of these differences varied among both men and women. All in all, however, the results suggest that the inequalities in self-rated general health observed with regard to educational level remained largely consistent over time. This is also in line with the results of the MONICA/KORA study, which focused on 25- to 64-year-olds in the Augsburg region in Germany. In terms of self-rated general health, this study identified marked differences across levels of education and income, which hardly changed from the beginning of the observation period (1984/1985) and over the following 15 years. However, whereas slight increases in both absolute and relative inequalities were observed, they did not reach statistical significance [26].

Furthermore, some further analyses exist that are based on SOEP data, the majority of which point to an increase in social inequalities in self-rated general health. For example, although an analysis of data on the working-age population (aged 18 to 59) found a significant deterioration in the general health of unemployed men between 1994 and 2008, it did not identify any significant changes among part-time and full-time working men. In the same manner, an increase in the initial difference between these groups was observed. Unemployed women also tended to provide a worse self-rating of their general health than women in part-time or full-time employment; however, the study found no evidence of a change in these differences over time [27, 28]. These findings are confirmed by a study based on SOEP data from 1994 to 2011, which showed that the span of income-related inequalities in general health have widened significantly among 25- to 60-year-olds [29]. The results of the econometric decomposition analysis conducted in this study indicate that the general rise in income inequality and the increased poverty rates among the unemployed are important factors that explain the widening gap in health inequalities between income groups.

A further study based on SOEP data collected between 2001 and 2011 examined the temporal development of self-rated general health in relation to material deprivation. Data on material deprivation was obtained using eleven standard items ranging from the ownership of a colour TV, a car and home furniture to the home environment, financial reserves and the ability to pay bills on time. This data suggests that the differences in self-rated health between people with a low and high material standard of living have increased between 2001 and 2005; by contrast, a reduction was observed for 2006 to 2011 [30].

Only limited conclusions can be deduced about time trends regarding social inequalities in the prevalence of chronic diseases in Germany. One of the few available studies uses data from the RKI’s health surveys to analyse time trends in educational differences in the prevalence of diabetes mellitus among 25- to 69-year-olds. The study found a significantly higher prevalence of known diabetes among women and men with low levels of education, compared to those with middle and high levels of education during both observation periods (1990 to 1992 and 1997 to 1999). However, the extent and pattern of these inequalities remained largely consistent over time [31]. A follow-up study considered additional data collected between 2002 and 2005. In regard to the time period from 1997 to 1999 and 2002 to 2005, the data indicates an increased prevalence of known diabetes among all education level groups. No significant shifts were found in terms of the educational gradient in the prevalence of known diabetes, as was the case with the previous study covering 1990 to 1992 and 1997 to 1999 [32].

A further analysis focused on time trends in social inequalities in cardiovascular diseases among 40 to 79-year-olds, also based on data from the RKI’s health surveys. The results show a slight decrease in the prevalence of major cardiovascular diseases (myocardial infarction, coronary heart disease, congestive heart failure or stroke) among women for the observation period 1997 to 1999 and 2008 to 2011; no significant change was observed among men. When the data was stratified by socioeconomic status, the study identified a slightly higher prevalence among women and men with a low socioeconomic status than among those with a higher socioeconomic status. No temporal change in the socioeconomic differences in the prevalence of major cardiovascular disease was observed among women. As such, although a general decline in prevalence was observed among all socioeconomic groups, the differences between the socioeconomic groups remained largely unchanged. In contrast, a widening in the range of these socioeconomic inequalities was observed among men. A decrease in prevalence was observed among men with a high socioeconomic status, but remained unchanged among the other socioeconomic groups [33].

SOEP data can be used to analyse time trends in inequalities in the prevalence of physical pain. Between 1994 and 2014, there was a slight increase in the proportion of women and men aged 25 to 69 who were always or often afflicted by physical pain during the four weeks preceding the survey. This increase occurred to a similar extent across all income groups. Consequently, the extent of these health inequalities has remained largely constant over time.

A further study based on SOEP data, which investigates the development in subjective health-related quality of life among 30- to 49-year-olds [25], came to a similar conclusion. The study assessed health-related quality of life using the Short Form-12 Health Survey (SF-12), a questionnaire widely used in epidemiological research. The study found that differences in the perceived limitations that people face in their day-to-day activities or at work due to their physical condition remained relatively consistent between 2002 and 2014 for the groups with a middle and high level of education, who scored more favourably compared to the group with the lowest level of education.

**Figure 2:**
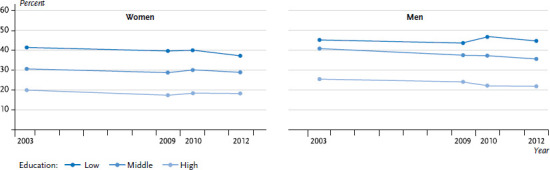
Time trends in smoking prevalence among women and men aged 25 to 69 years by educational level (age-standardised to the 2013 European standard population) Source: GSTel03, GEDA 2009, GEDA 2010, GEDA 2012 [34]

Two recent studies provide information about developments over time regarding social inequalities in people’s health-related behaviour. These are based on data from the German Health Update (GEDA 2009, 2010 and 2012) and the German Telephone Health Survey 2003 (GSTel03) that preceded it [34, 35]. The first of the two studies focuses on whether the decline in tobacco use, which began around 2003, is common to all educational levels, or is only or particularly prevalent among groups with higher levels of education. The GEDA data shows that men aged between 25 and 69 years with a medium or high level of education smoked less frequently in 2012 than ten years previously. In contrast, no such decline in the proportion of smokers was observed among men with lower levels of education. In regard to tobacco consumption, therefore, the range of educational inequalities widened over the observation period. Moreover, a decline in tobacco consumption was detected among women, whereas differences according to educational level remained relatively constant over time (Figure 2).

**Figure 3:**
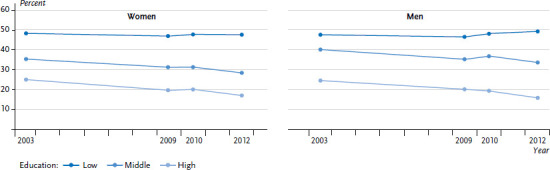
Time trends in sporting inactivity among women and men aged 25 to 69 years by educational level (age-standardised to the 2013 European standard population) Source: GSTel03, GEDA 2009, GEDA 2010, GEDA 2012 [35]

The second study focuses on changes in educational differences in sporting activity [35]. As with smoking, a positive overall trend was identified for participation in sporting activity. This can be surmised from the clear decline in the proportion of women and men who do not participate in sport at all. This development can be seen among women and men in both the groups with middle and higher levels of education. On the other hand, the proportion of women and men with a low level of education who do not engage in sport remained largely unchanged during the observation period. These trends led the gap in sporting inactivity between the educational groups to widen between 2003 and 2012 (Figure 3).

**Figure 4:**
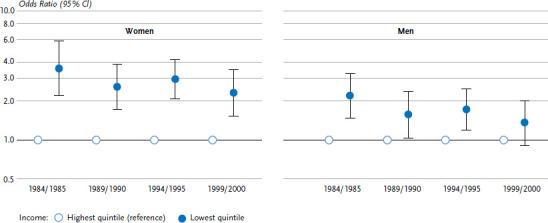
Time trends in relative income inequalities in obesity among women and men aged 25 to 64 years in the Augsburg region (Germany) Source: MONICA/KORA-Surveys 1984/1985 to 1999/2000 [26]

Studies have also been published on time trends in social inequalities in the prevalence of overweight and obesity [26, 36, 37]. In most cases, these studies focused on body mass index – the ratio of a person’s weight to the square of their height (kg/m²). They suggest that the increase in obesity observed between the mid-1980s and the beginning of the 2000s was not confined to socially disadvantaged groups [26, 36]. For example, results from the Augsburg MONICA/KORA study emphasise that the prevalence of obesity increased among 25- to 64-year-old women and men in almost all levels of education and income groups [26]. Moreover, absolute inequalities between educational level and income groups have barely changed. On the other hand, relative inequalities have decreased somewhat, as illustrated by Figure 4, which compares the fifth of the population with the lowest income to the fifth with the highest income level.

Results regarding social inequalities in mortality and life expectancy play a central role in the scientific and political discussion of health inequalities [15]. Only a few studies have analysed the temporal developments of these differences for Germany. One such study based on SOEP data examines changes in the income-related and educational differences in healthy life expectancy between 1989, 1999 and 2009. A healthy life expectancy is defined as the subjective satisfaction with the subject’s own health. The study shows that social inequalities in healthy life expectancy have widened over the observation period, especially among men. This increase has been linked to the widening gap between income and educational groups in terms of people’s subjective satisfaction with their own health. Social inequalities in mortality, on the other hand, have remained largely consistent over time [38].

**Figure 5:**
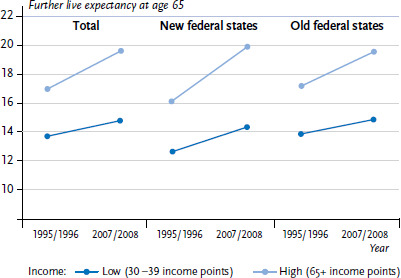
Time trends in further life expectancy at age 65 years among men with statutory pension insurance by income level Source: DRV Bund [39]

Data from the German Statutory Pension Insurance Scheme (DRV) was used to analyse developments over time in regard to social inequalities in further life expectancy from the age of 65 years. Differences in terms of income level (earnings points) and occupational status were examined over an observation period from 1995/1996 to 2007/2008. The study found that social inequalities in further life expectancy have increased (Figure 5). Although life expectancy has increased in all of the groups under study, this increase was less prominent among groups with lower incomes and lower occupational positions. As a result, during the observation period, the social gap in life expectancy widened by 1.7 years between income groups (those with 30 to 39 compared to 65 and over earnings points) and 0.9 years between occupational groups. This development was more pronounced in the new German federal states [39].

**Figure 6:**
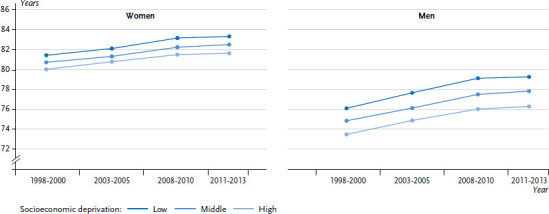
Time trends in life expectancy at birth by regional socioeconomic deprivation at the district level Source: Indicators and Maps on Spatial and Urban Development (INKAR) [40]

The relationship between social inequality and health or life expectancy can be determined at both the individual and the regional level. For example, it has been shown repeatedly at the regional (defined as the spatial planning regions designated by the Federal Institute for Research on Building, Urban Affairs and Spatial Development – BBSR), the district and municipal level that regions with a higher risk of poverty or higher levels of unemployment have lower average life expectancies [15]. In order to analyse time trends in this field, a newly developed index of socioeconomic deprivation at the regional level has recently been used. Many indicators from the fields of employment, education and income were taken into account during the development of the German Index of Socioeconomic Deprivation (GISD) [40]. With the help of the GISD it could be shown that, from 1998-2000 to 2011-2013, the mean life expectancy of men in advantaged districts was 2.9 years longer than the mean life expectancy of men living in the most deprived districts. Among women, this difference in mean life expectancy was 1.5 years. The extent of regional socioeconomic inequalities in life expectancy increased significantly during the observation period (27.7 % among women and 20.2 % among men). Expressed in years, the difference in life expectancy between districts with high and low levels of socioeconomic deprivation has risen from 1.4 to 1.7 years among women, and from 2.6 to 3.0 years among men (Figure 6).

## 3. International research

In the UK, the US and Scandinavia, analyses of social inequalities in health and life expectancy have been possible for quite some time. As in Germany, self-rated general health is a commonly used indicator. Most of the existing studies conclude that social inequalities in overall health have remained largely stable over the past two to three decades or have increased slightly [41-47]. In a recent study, data from 17 European countries were evaluated for the period ranging from 1990 to 2010 [42]. In almost all countries, data from the last available study year showed that the proportion of women and men who rated their health as less than good was much higher in the group with low education and among manual labourers than among those with the highest levels of education and people working in non-manual occupations. When the data from all of these countries were considered in a meta-analysis, the data showed that although the overall proportion of people who rated their health as less than good decreased during the observation period, the health gaps between the educational and occupational groups either remained the same (absolute inequalities in educational level among men; absolute occupational inequalities in both sexes) or actually increased (absolute inequalities in education among women; relative inequalities in educational and occupational groups in both sexes).

**Figure 7:**
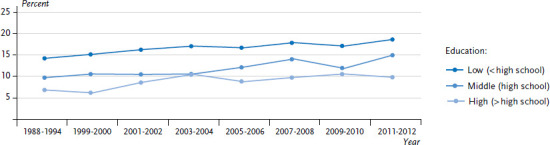
Time trends in diabetes prevalence among people aged 20 years or above in the US by educational level Source: National Health and Nutrition Examination Survey (NHANES) 1988-2012 [48]

Findings on the temporal development of social differences in the prevalence of diabetes mellitus are available, for example, from the United States [48]. Data from the National Health and Nutrition Examination Survey (NHANES) demonstrates a significant increase in the prevalence of diabetes, which rose from 9.8 % to 12.4 % between 1988 and 2012. A clear social gradient was identified for education at both the beginning and the end of this time period, with the prevalence of diabetes increasing with declining levels of education. However, there was a significant increase in the prevalence of diabetes over time among all educational groups, meaning that no significant change was identified in the differences between the groups (Figure 7).

Social inequalities in serious health conditions and diseases such as heart attacks and strokes [49-51], some of which are particularly pronounced, are also reported to have remained relatively consistent over time. In addition, findings on trends in social inequalities in mental health are also available for several European countries. For example, an increase in the social gradient in mental health problems has been reported for England. This has been attributed to a particularly strong increase in mental health problems among lower socioeconomic groups from the end of 2008, a period during which structural changes took place in the country’s labour market and welfare state reforms were implemented following the global financial crisis [52]. Different findings have been published on developments in social inequalities in mental health in Scandinavia. On the one hand, register-based studies from Finland indicate stable occupational class differences in sickness-absence at work due to mental disorders; on the other hand, they also suggest an increase in educational inequalities in psychiatric hospitalisation rates [53, 54].

The results on time trends in social inequalities in functional limitations are also interesting. For example, an analysis of data collected by the European Social Survey for elderly people from 16 European countries shows that the prevalence of functional limitations decreased in most countries between 2002 and 2014. In the course of these developments, absolute and relative inequalities in the prevalence of functional limitations between different income groups have increased, which is particularly apparent in Ireland, the Netherlands and Sweden [55]. Similar findings are also available for the elderly population in the US, albeit for an earlier observation period ranging from 1982 to 2002 [56].

With regard to tobacco consumption, international studies concur with the results obtained for Germany: smoking is more prevalent and heavily practised among lower socioeconomic groups [57, 58]. Moreover, these differences have either remained stable over the past two to three decades or have increased [57-62]. Whereas differences in smoking habits among 25- to 79-year-old men from seven Western European countries remained virtually unchanged between 1985 and 2000, there was a significant increase in absolute and relative educational inequalities in smoking among women of the same age [57]. Studies based on more recent data, for example from the Netherlands [63] and Australia [64], also suggest an increase in social inequalities in smoking since the early 2000s as a result of a general decline in smoking prevalence.

In many countries, physical activity is more prevalent among socially advantaged groups than among lower socioeconomic groups [61, 64-66]. Some of the available studies on temporal developments in social inequalities in leisure-time physical activity report that inequalities have increased over time [62, 67]. However, others suggest that these differences have remained stable [64, 68]. A US study across all 50 states found that between 1990 and 2004, educational inequalities in leisure-time physical inactivity increased in 31 states, whereas a reduction was observed in only seven states [62].

The same study also analysed time trends in educational inequalities in obesity. However, in this case, the study identified a reverse trend: between 1990 and 2004, inequalities in obesity increased in only six states, whereas they declined in 36 others. This was mostly due to a stronger increase in the prevalence of obesity in the groups with higher levels of education over the observation period [62]. According to a recent study, which evaluated data from 15 European countries, the overall prevalence of obesity among 30- to 64-year-old women and men increased between 1990 and 2010 [69]. This increase was more pronounced among women with lower levels of education than among women with higher levels of education. Similarly, the average increase in obesity prevalence among men was greater in the group with a lower level of education than in the group with a higher level of education. An analysis of the data from all included countries demonstrates that absolute educational inequalities in obesity increased significantly during the observation period (around 20 years), whereas relative inequalities persisted or even decreased slightly during the same period. Trend analyses from Austria [70], France [71] and Switzerland [72] suggest that educational inequalities in obesity have either stabilised or widened.

In comparison to Germany, the data for analysing time trends in health inequalities is far superior in many other countries. This is particularly true for data on mortality and life expectancy. In some countries, information concerning socioeconomic status, such as educational level and occupational position, is even recorded on death certificates – a practise that has been discontinued in Germany since 1972 [73, 74]. In addition, some countries keep mortality registers, and the data they hold can be linked to other registry, census or administrative data [18, 75]. A fairly long time series of data is available for England and Wales on inequalities in mortality. The data demonstrates that social inequalities in mortality initially decreased from the 1920s until the early 1950s – that is, during the early years of the British welfare state [3, 76]. After this point, they begin to increase once again. Data from the UK, Scandinavian and Eastern European countries suggests that social inequalities in mortality probably increased further towards the end of the 20th century [17, 77-79]. In most countries, these developments reflect the trend that higher socioeconomic groups have benefited more in recent decades from the decline in premature mortality and rising life expectancy than lower socioeconomic groups. This is also confirmed by a recent study based on data from 17 European countries for 1980 to 2010 [80]. The results show that all-cause mortality among groups with a high level of education has fallen by an average of 2.5 % per year, whereas the annual decline among groups with middle and lower levels of education has been lower, at 1.8 % and 1.3 % respectively.

**Figure 8:**
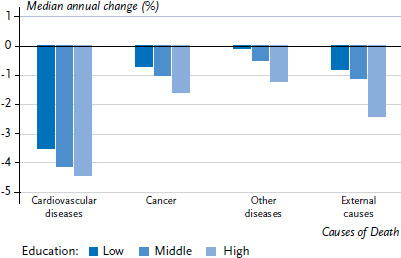
Median annual change in mortality by grouped causes of death and educational level in 17 European countries between 1980 and 2010 Source: Mackenbach et al. 2017 [80]

Looking at the mortality trends by cause of death, a more pronounced reduction in mortality can be observed among higher educational groups due to cardiovascular diseases and cancer, but also mortality due to other diseases (such as tuberculosis and asthma) and external causes of death (such as suicide and homicide) (Figure 8). Alcohol-related causes – subsumed under ‘other diseases’ in Figure 8 – are the only specific causes of death which are associated with an increased rather than decreased mortality in many European countries [80, 81]. For example, alcohol-related mortality among low soci-oeconomic groups has increased particularly rapidly in some Eastern and Northern European countries, and this has led social inequalities in alcohol-related mortality to increase significantly, to the disadvantage of low socioeconomic groups [81].

Breast cancer mortality also plays a special role in the specific causes of death and their social patterning. In contrast to the social gradient observed for most other causes of death, until the 1990s a higher level of breast cancer mortality existed internationally among high socioeconomic groups [82, 83]. Recent European data suggests that this relationship has decreased significantly and has even undergone a significant reversal since the early 2000s, to the extent that today, breast cancer mortality is higher in low socioeconomic groups [84]. One possible reason for this ‘turn of the gradient’ is that women in higher socioeconomic groups may have benefited more from improvements in early detection and breast cancer treatment over the past few decades than women in low socioeconomic groups, and thus their chances of survival may have improved disproportionately. In addition, social inequalities in trends in breast cancer risk factors that are related to reproductive behaviours (such as breastfeeding behaviour, age at first birth, use of contraceptives and hormone replacement therapy) or lifestyle (alcohol consumption, physical activity) could also play a role [84].

Studies that systematically differentiate between absolute and relative social inequalities in mortality show that health inequalities can increase and decrease during the same time period, depending on whether relative inequalities (rate ratios) or absolute inequalities (rate differences) are taken into account [16, 18]. For example, an analysis of data from six European countries between the 1980s and the 1990s found an increase in relative inequalities in all-cause mortality, whereas absolute inequalities remained fairly constant [17]. Another study, based on data from 11 European countries, indicated that in some countries relative inequalities in mortality increased between 1990 and 2010, as the percentage reduction in mortality rates in low educational groups was lower than in higher educational groups [18]. On the other hand, the absolute reduction in mortality rates was sometimes lower among higher educational groups, so that absolute inequalities in educational levels in some countries decreased; this was particularly evident among men (Table 1).

Social inequalities in mortality are also reflected in social inequalities in life expectancies, so that time trends correspond closely with one another. Individual studies from Europe indicate that individuals from all socioeconomic groups have benefited from the general increased life expectancy in countries such as Denmark, Norway and the Netherlands; however, people in high socioeconomic groups have benefited more in terms of additional years of life expectancy compared to low socioeconomic groups [85-87]. As a result, social inequalities in life expectancy have increased in these countries. Other studies from Belgium, Sweden and Finland point to a more stagnant or even declining rate of life expectancy among low income groups [88] and among women with lower levels of education [89, 90]. At the same time, life expectancy among people in higher socioeconomic groups increased, which has also led to a widening of inequalities in life expectancy. At the same time, there has been a reduction in social inequalities in life expectancy since the 1980s among women in Austria and Italy, although this trend has not been observed to an equal extent among men [91, 92]. For the United States, a very comprehensive study has recently been published, indicating a substantial widening of social differences in life expectancy since the early 2000s [75]. The study analysed administrative data from 1.4 billion tax and death records. The results show that between 2001 and 2014, life expectancy increased by 2.91 years for women and 2.34 years for men in the top 5 % of the income distribution, but only by 0.04 and 0.32 years for those in the bottom 5 %.

## 4. Discussion

Every year, a large number of studies demonstrate that substantial social inequalities continue to exist in health and life expectancy in Germany and other countries. Since the data available at the national level has been steadily improving, it is now possible to study time trends in health inequalities in Germany. The studies undertaken so far either suggest that social inequalities in health and life expectancy in Germany have remained relatively constant over the last 20 to 30 years, or that they have increased.

It is important to note that the results vary according to the respective socioeconomic indicator and health outcome used in each study, and according to the age group examined. Moreover, inconsistent results have sometimes been obtained from the same data. Most of the studies on trends in health inequalities in Germany examined self-rated general health, with a particular focus on SOEP data. The results suggest that although general health has improved over time, this has only been the case among the socially advantaged groups; moreover, strongly pronounced health inequalities tend to have increased further. The majority of these studies compare educational level and income groups. However, one study that compared unemployed people to parttime and full-time employees came to the same conclusions regarding these developments. However, the studies that focused on material deprivation do not quite fit this picture. An increase in inequalities in self-rated general health was reported between 2001 and 2005; however, it was followed by a decrease in the following years.

Very few analyses are currently available for Germany when it comes to time trends in social inequalities in chronic diseases. The only available studies focus on diabetes mellitus and cardiovascular diseases. The results indicate that social inequalities remained relatively stable over time. Only one study reported an increase in social inequalities in cardiovascular diseases among men, which was attributed to the observed decline in cardiovascular diseases among men with a high socioeconomic status. In addition, the available studies on chronic pain and physical impairment related to health-related quality of life indicate that pronounced social inequalities continue to exist and have hardly changed over time.

Health inequalities have also widened in terms of health behaviours and behavioural risk factors. This is particularly clear in relation to sporting activity, as the proportion of women and men who do not engage in any form of sport has clearly declined in the middle and high educational groups, and has remained largely unchanged among the lower educational group. With regard to men’s use of tobacco, positive developments such as the decline in smoking rates observed since the early 2000s have only been found among socially advantaged groups.

Finally, the few available studies addressing developments in social inequalities in mortality and life expectancy in Germany suggest that health inequalities have widened rather than decreased. This is the case on both the individual and regional level. By the late 1990s, people living in districts that can be considered as socioeconomically advantaged in terms of the labour market, education and income had higher life expectancies at birth than those living in socioeconomically deprived districts. In recent times, these inequalities have increased further.

The results achieved for Germany largely confirm the findings of the latest international research. Studies from the US, the UK and Scandinavia are particularly good examples. The superior data in these countries enables trends in health inequalities to be analysed over long periods of time. Most of the studies carried out in these countries show that, in regard to self-rated health, certain diseases and behavioural risk factors, social inequalities are repeatedly reproduced even over long periods of time and under changed framework conditions. In some cases social inequalities are increasing – for example, when a risk or prevalence rate among socially advantaged population groups declines faster and more rapidly than among socially disadvantaged groups. This phenomenon can be observed for tobacco consumption in many countries. Studies of the temporal development of social inequalities in mortality demonstrate that conclusions about whether social inequalities have diminished or increased may also depend on whether absolute or relative inequalities are being considered. The results from the US are remarkable, as they show that high income groups have registered a two- to three-year increase in life expectancy since the beginning of the 2000s, whereas people at the bottom end of the income scale have seen no significant increase in life expectancy whatsoever [75].

Against the background of these national and international research findings, the reduction of health inequalities clearly remains an important policy objective. In this respect, a distinction can be made between measures aimed at improving and ensuring fairer social conditions and opportunities for social and economic participation – that is, measures that seek to tackle the underlying causes of health inequalities – and those that seek to directly improve health and to bring about a fairer distribution in opportunities for good health. On the one hand, this leads to a focus on measures aimed at combating poverty and social inequality as well as the strengthening of social integration; these measures need to be incorporated into various policy areas. The measures in question are far more comprehensive than simple state transfer benefits, which, by themselves, are not sufficient to guarantee people equal social participation. Moreover, these measures must also aim to improve people’s participation in education, reduce inequalities in educational opportunities, improve opportunities in the labour market for low-skilled workers, support and empower socially disadvantaged families, and reduce environmental pressures and risks that increasingly affect socially disadvantaged groups [11, 93].

On the other hand, health policy needs to take action, not only in terms of medical and nursing care, but particularly in prevention and health promotion. In the context of medical and nursing care, it is important to note that socially disadvantaged groups have a greater need for health care due to their increased risk of chronic illnesses, disabilities and functional limitations. In addition, they also often have specific health care needs, for example due to multi-morbidities, mental co-morbidities or addiction. Finally, social disadvantaged people do not have the same financial capabilities to access medical services and goods that are not covered by the statutory health insurance [94].

Prevention and health promotion are viewed as particularly important factors that can reduce health inequalities [8, 95]. Counteracting the risk of early morbidity and mortality among socially disadvantaged groups is a particularly effective way of reducing health inequalities. However, doing so requires preventive and health-promoting measures that include and particularly target the socially disadvantaged. Therefore, these measures need to take effect at the earliest stages possible – at birth and during early childhood and adolescence. In this regard, interventions aimed at clearly defined population groups, which take into account the living conditions, settings and problems of the respective population groups and involve the target groups and relevant institutions and actors in the stages of planning, implementation and evaluation of the measures, have proven to be particularly effective [96, 97].

Despite positive developments, such as the adoption of the Preventative Health Care Act (PrevG) which came into force in 2015 [98] and the continuity of cooperation networks such as Equity in Health (cooperation network ‘Gesundheitliche Chancengleichheit’) [99] or Germany’s national health goals (www.gesundheitsziele.de) [100], Germany still lacks a comprehensive policy strategy aimed at reducing health inequalities. Countries such as the UK [101, 102] and Sweden [103] are further ahead on this issue. In addition to their focus on priority public health issues, the programmes implemented in these countries are supported by several ministries as part of a ‘health in all policies’ approach, and involve key stakeholders at different levels. In addition, they have been extensively documented and even partially evaluated; however, the results of these evaluations have often indicated that the measures implemented so far are still not sufficient. For example, evaluations of the ‘English strategy’, a 13-year programme by the English government aimed at reducing health inequalities, demonstrate that the programme has yet to achieve any lasting reduction in social inequalities in terms of health and life expectancy [104-106]. However, the importance of such policy programmes is not generally questioned. Rather, the strategy’s shortcomings are explained by the fact that it does not address the most relevant entry-points, did not use effective policies and was not delivered at a large enough scale for achieving population-wide impacts [105]. Moreover, political reforms implemented towards the end of the 13-year programme – such as cuts to social benefits and public expenditure as a result of the financial and economic crisis – may have at least partially reversed the achievements of the strategy [104].

Experiences gained from countries such as the UK and Sweden show that reducing health inequalities is a task for the whole of society. It requires cross-sectorial determination that is focused on tackling the root causes (to a large extent, these are found in underprivileged living conditions and reduced opportunities for social participation), and highlights the importance of anchoring prevention and health promotion within all policy areas. Finally, experience has shown that reducing health inequalities is a long-term task, and that successful measures require a stable structural and legal framework as well as secure funding.

## Key statements

- Low socioeconomic status is associated with a higher risk of morbidity and premature mortality.
- In many respects, the extent of health inequalities has remained constant over the past 20 to 30 years.
- Increases in health inequalities can be observed, for example, with regard to sporting activity and life expectancy.
- The results for Germany largely correspond to the latest international findings.
- Reducing health inequalities is an important health policy objective and requires efforts on the part of society as a whole.

## Figures and Tables

**Table 1:** Rate differences and rate ratios of all-cause mortality (comparing low versus high educational group) among women and men in 11 European countries in 1990-1994 and 2005-2009 Source: Mackenbach et al. 2016 [18]

	Absolute inequalities (rate differences^a^)			Relative inequalities (rate ratios)		
	1990-1994	2005-2009	*p*-trend	1990-1994	2005-2009	*p*-trend
**Women**						
Finland	231	277	<0.001^*^	1.46	1.78	< 0.001^*^
Sweden	248	266	0.055	1.62	1.80	< 0.001^*^
Norway	328	392	<0.001^*^	1.65	2.05	< 0.001^*^
Scotland	335	307	0.755	1.57	1.75	0.521
England and Wales	254	193	0.120	1.46	1.46	0.944
France	208	214	0.885	1.62	1.76	0.548
Switzerland	171	170	0.984	1.39	1.53	0.015*
Spain (Barcelona)	179	127	0.005^*^	1.49	1.45	0.640
Italy (Turin)	141	109	0.346	1.33	1.37	0.724
Slovenia	248	251	0.971	1.47	1.76	0.809
Lithuania	93	765	< 0.001^*^	1.17	3.24	< 0.001^*^
**Men**						
Finland	658	667	0.562	1.66	2.06	< 0.001^*^
Sweden	445	412	0.010^*^	1.60	1.78	< 0.001^*^
Norway	711	688	0.340	1.75	2.15	< 0.001^*^
Scotland	681	502	0.030^*^	1.81	1.83	0.521
England and Wales	494	317	< 0.001^*^	1.55	1.57	0.944
France	677	574	0.069	2.00	2.00	0.548
Switzerland	688	557	< 0.001^*^	1.86	2.10	0.015^*^
Spain (Barcelona)	552	412	< 0.001^*^	1.64	1.71	0.640
Italy (Turin)	384	340	0.242	1.47	1.70	0.724
Slovenia	814	806	0.086	1.85	2.31	0.809
Lithuania	569	1722	< 0.001*	1.56	2.89	< 0.001^*^

^a^ deaths per 100,000 person-years

* significant (*p* < 0.05)
